# Dynamic Magnonic Crystals Based on Spatiotemporal Plasmon Excitation

**DOI:** 10.1002/adma.202502474

**Published:** 2025-06-03

**Authors:** Nikolai Kuznetsov, Huajun Qin, Lukáš Flajšman, Sebastiaan van Dijken

**Affiliations:** ^1^ NanoSpin Department of Applied Physics Aalto University School of Science Aalto FI‐00076 Finland; ^2^ School of Physics and Technology Wuhan University Wuhan 430072 China; ^3^ Wuhan Institute of Quantum Technology Wuhan 430206 China

**Keywords:** dynamic magnonic crystals, magnonics, metamaterials, plasmonics

## Abstract

Metamaterials, designed to exhibit properties beyond those found in nature, enable unprecedented control over physical phenomena through flexible band structure engineering. This work introduces a hybrid magnonic‐plasmonic metamaterial that allows spatiotemporal manipulation of spin‐wave transport at micrometer scales and sub‐microsecond timescales. The system integrates a plasmonic metamaterial, comprising Au nanodisk arrays arranged in a 1D periodic stripe pattern, with a low‐damping yttrium iron garnet (YIG) film as the spin‐wave transport medium. Short laser pulses (100−500 ns) excite surface lattice resonances (SLRs) in the plasmonic stripes, inducing thermoplasmonic heating and generating a striped temperature profile. This dynamic thermal modulation periodically alters the YIG film's saturation magnetization, forming a laser‐controlled magnonic crystal. Time‐resolved propagating spin‐wave spectroscopy reveals tunable bandgaps and minibands arising from Bragg reflection. By adjusting the plasmonic stripe pattern, laser pulse duration, or power, this system enables precise control over spin‐wave transport, paving the way for reconfigurable wave‐based computing devices.

## Graphical abstract

Hybrid magnonic‐plasmonic metamaterials enabling spatiotemporal optical control of spin‐wave transport are demonstrated. These structures integrate periodic stripes of gold nanodisk arrays with low‐damping yttrium iron garnet films to realize dynamically reconfigurable magnonic crystals. By utilizing localized thermoplasmonic heating, induced by short laser pulses, the system achieves active modulation of the magnonic band structure, including the formation of bandgaps and minibands.

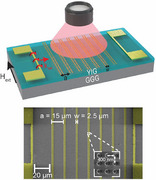

## 1 Introduction

Metamaterials are artificially engineered materials designed to exhibit properties not typically observed in natural materials. Their behavior is primarily governed by structural parameters, such as size, shape, periodicity, and orientation, rather than chemical composition. By tailoring these parameters, it is possible to manipulate electromagnetic waves,^[^ ^1^ ^]^ sound waves,^[^ ^2^ ^]^ mechanical forces,^[^ ^3^ ^]^ and other physical phenomena through precise band structure engineering. Recent advances have enabled the development of dynamically tunable metamaterials,^[^ ^4^ ^]^ whose properties can be actively controlled in real time. This is achieved through external stimuli such as electric or magnetic fields, optical pumping, thermal modulation, or mechanical actuation.

In plasmonics, which explores the interaction between electromagnetic waves and free electrons on metal surfaces, metamaterials are used to control light at scales smaller than its wavelength. A notable example is the plasmonic lattice, an array of metal nanostructures. When light interacts with the free electrons in these nanostructures, it generates collective oscillations known as localized surface plasmon resonances (LSPRs).^[^ ^5^ ^]^ These LSPRs can couple to form surface lattice resonances (SLRs).^[^ ^6^, ^7^ ^]^ The excitation of an SLR mode enhances light absorption, a property utilized in plasmonic biosensors to detect subtle environmental changes^[^ ^8^ ^]^ and in photovoltaic cells to improve efficiency.^[^ ^9^ ^]^ Additionally, this optical absorption can induce thermoplasmonic heating,^[^ ^10^ ^]^ enabling the generation of spatiotemporal heating patterns.

Magnonics is a branch of metamaterials research dedicated to controlling coherent spin excitations in magnetic materials, known as spin waves or magnons. In this field, metamaterials are referred to as magnonic crystals,^[^ ^11^, ^12^, ^13^ ^]^ which are engineered structures with periodic variations in one or more magnetic parameters. Examples include 1D magnetic stripe arrays,^[^ ^14^ ^]^ magnetic antidot lattices,^[^ ^15^ ^]^ 2D arrays of magnetic nanoelements,^[^ ^16^ ^]^ and magnetic stripe domains.^[^ ^17^, ^18^ ^]^ These structures modify spin‐wave propagation via Bragg reflection, forming magnonic band structures with well‐defined bandgaps and minibands. Magnonic crystals have been integrated into various microwave devices, including filters,^[^ ^19^ ^]^ phase shifters,^[^ ^20^, ^21^ ^]^ magnon transistors,^[^ ^22^ ^]^ and magnetic field sensors.^[^ ^23^ ^]^ They also play a pivotal role in magnonic wave‐based computing,^[^ ^24^, ^25^, ^26^, ^27^ ^]^ an emerging paradigm for energy‐efficient information processing that relies on the manipulation of spin‐wave amplitude and phase. A key breakthrough in this field has been the development of dynamic magnonic crystals, which enable active control of spin waves in both space and time. These have been realized through magnetic switching,^[^ ^14^ ^]^ laser pulsing,^[^ ^28^, ^29^ ^]^ and electric currents.^[^ ^30^, ^31^, ^32^ ^]^ However, scalability remains a challenge: magnetic switching requires bulky electromagnets, while approaches based on spatial light modulation or meandering current‐carrying wires are limited by their millimeter‐scale footprints.

Building on our previous work demonstrating active control of spin‐wave transport via thermoplasmonic heating in large plasmonic arrays,^[^ ^33^ ^]^ we now extend this concept by patterning the arrays into 1D periodic stripes to realize dynamic magnonic crystals. This approach enables optical control of spin waves on micrometer scales and sub‐microsecond timescales. Our system consists of a plasmonic metamaterial, Au nanodisk arrays patterned into 1D periodic stripes, integrated with a low‐damping yttrium iron garnet (YIG) film that supports spin‐wave propagation. Together, this hybrid structure forms an optically reconfigurable magnonic crystal. Spin‐wave transport is modulated by exciting an SLR mode in the plasmonic stripes using short laser pulses (100 − 500 ns). The resulting striped temperature profile in the YIG film induces a spatial modulation of its saturation magnetization. Using time‐resolved propagating spin‐wave spectroscopy, we demonstrate the formation of a laser‐induced magnonic band structure with multiple bandgaps and minibands. This band structure arises from Bragg reflection of propagating spin waves and can be tuned by adjusting the number, width, and period of the plasmonic stripes, or by varying the laser pulse duration and power.

## 2 Results

**Figure 1:**
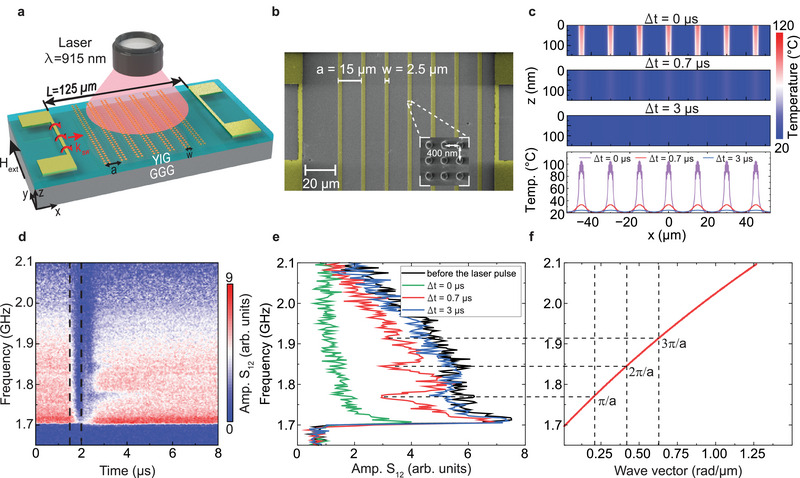
Spatiotemporal control of spin‐wave transport in a magnonic‐plasmonic metamaterial. a) Schematic of the sample and measurement geometry. The hybrid structure consists of arrays of Au nanodisks arranged in stripes on an 80‐nm‐thick YIG film. Single laser pulses (wavelength: 915 nm, duration: 100−500 ns, power: 3.3−16.6 W) excite an SLR mode in the plasmonic stripes, generating a striped temperature profile in the YIG film via thermoplasmonic heating. Two parallel microwave antennas patterned on the YIG film facilitate spin‐wave excitation and detection, enabling the study of thermoplasmonic heating effects on spin‐wave transmission through time‐resolved propagating spin‐wave spectroscopy. b) SEM image of the *a*15*w*2.5 magnonic‐plasmonic metamaterial with *N* = 7. c) Modeling of thermoplasmonic heating in the *a*15*w*2.5 structure under a 500 ns laser pulse (*P* = 16.6 W). The top panel shows thermal profiles across the YIG film in the *xz*‐plane at various times (Δ*t*) after the pulse is switched off. The bottom panel presents line scans of the temperature distribution at the YIG film's top interface at different Δ*t*. d) Time‐resolved map of spin‐wave transmission spectra for the *a*15*w*2.5 metamaterial, with the 500 ns laser pulse (*P* = 16.6 W) activated at *t* = 1.5 µs. The vertical dashed lines mark the laser pulse activation and deactivation times. e) Spin‐wave transmission spectra extracted from (d) at different Δ*t* values. f) Spin‐wave dispersion in the 80‐nm‐thick YIG film derived from SNS‐MOKE microscopy measurements. Horizontal and vertical dashed lines mark the center frequencies of the bandgaps and their corresponding wave vectors. The wave vectors satisfy the Bragg condition *k* = *n* *π*/*a*. All experiments and simulations were conducted with a 16 mT magnetic bias field aligned parallel to the microwave antennas.

**Figure** **1**a,b presents a schematic and scanning electron microscopy (SEM) image of the magnonic‐plasmonic metamaterial and the measurement geometry. The YIG film, 80 nm thick, was grown epitaxially on a (111)‐oriented gadolinium gallium garnet (GGG) substrate using liquid‐phase epitaxy (LPE). Plasmonic nanodisk arrays, consisting of a 3 nm Ti/50 nm Au structure, were patterned on the YIG film using electron‐beam lithography and lift‐off techniques. We selected the disk diameter (*d* = 180 nm) and array period (*p* = 400 nm) to maximize light absorption at the 915 nm wavelength used in the spin‐wave transmission experiments (see Figure S1, Supporting Information for optical transmittance data).^[^ ^33^ ^]^ The nanodisk arrays (inset in Figure 1b) were arranged in stripe patterns with stripe widths (*w*) ranging from 1 to 5 µm. We also varied the stripe period (*a*) and stripe count (*N*) to examine their effects on the optical control of spin‐wave transport.

Spin‐wave transport beneath the plasmonic stripes was characterized using time‐resolved propagating spin‐wave spectroscopy. In this technique, spin waves are excited by a microwave antenna and detected inductively by a parallel antenna, with a vector network analyzer (VNA) recording spin‐wave transmission through the *S*_12_ scattering parameter. To facilitate these measurements, we patterned microwave antennas on either side of the plasmonic stripes using photolithography (Figure 1a,b). The antennas, measuring 6 µm in width and 50 µm in length, were separated by 125 µm. The 250‐µm‐long plasmonic stripes were designed to span the region between the antennas, ensuring that propagating spin waves passed through them. A magnetic bias field of 16 mT was applied parallel to the antennas to facilitate spin‐wave transport in the Damon‐Eshbach (DE) geometry, where the wave vector is oriented perpendicular to the in‐plane magnetization of the YIG film.

Optical control of propagating spin waves was achieved by integrating a 915 nm pulsed laser diode into the spin‐wave spectroscopy setup (Figure 1a). We focused the laser onto the plasmonic stripes with a spot diameter of 70 µm. Pulse durations were varied from 100 to 500 ns, while the laser power was adjusted between 3.3 and 16.6 W to investigate their influence on spin‐wave dynamics. To monitor spin‐wave transport under optical excitation, the VNA and pulsed laser were synchronized using an external waveform generator. Time‐resolved optical pump‐microwave probe measurements were performed by varying the VNA measurement‐point delay relative to the trigger signal across multiple frequencies (see Experimental Section for details). Thermoplasmonic heating in the YIG film was evaluated using finite‐difference time domain (FDTD) simulations and thermal modeling in Lumerical software. The simulated temperature distribution in the YIG film following a 500 ns laser pulse is shown in Figure 1c. Immediately after the pulse (Δ*t* = 0 µs), the temperature beneath the plasmonic stripes rises to approximately 100°C, while the interstripe regions remain minimally heated. The optical transparency of YIG at 915 nm ensures that heating is primarily driven by SLR mode excitation within the plasmonic stripes. Over several microseconds of cooling, lateral heat diffusion broadens the temperature profile, though the periodic modulation persists throughout the cooling process (see Figure S2, Supporting Information for additional thermoplasmonic simulations).

Figure 1d,e presents time‐resolved spin‐wave spectroscopy measurements for a magnonic‐plasmonic metamaterial with a stripe period of *a* = 15 µm and stripe width of *w* = 2.5 µm (referred to as *a*15*w*2.5). In this experiment, a 500 ns laser pulse initiated at *t* = 1.5 µs leads to a significant reduction in spin‐wave transmission between the antennas, as indicated by the time evolution of the *S*_12_ parameter. At the end of the laser pulse (*t* = 2.0 µs, Δ*t* = 0 µs), when the temperature in the YIG film beneath the plasmonic stripes reaches its peak, the spin‐wave signal is attenuated strongly across the entire spectrum, with no detectable bandgaps (green curve, Figure 1e). After a cooling period of Δ*t* = 0.7 µs, three bandgaps have formed in the spin‐wave transmission spectrum (red curve, Figure 1e), and the signal intensity within the minibands approaches that of the reference spectrum recorded before the laser pulse (black curve, Figure 1e). As cooling continues, the bandgaps gradually diminish, and no bandgaps are observed at Δ*t* = 3 µs (blue curve, Figure 1e). The laser‐induced magnonic bandgaps arise from spin‐wave Bragg scattering, induced by the periodic temperature modulation in the YIG film (Figure 1c). The spin‐wave dispersion in Figure 1f, derived from super‐Nyquist sampling magneto‐optical Kerr effect (SNS‐MOKE) microscopy measurements on the YIG film (Figure S3, Supporting Information), supports this interpretation. The bandgap frequencies observed at Δ*t* = 0.7 µs correspond to wave vectors *k* = *n* *π*/*a*, where *π*/*a* represents the first Brillouin zone boundary of the magnonic band structure, and *n* denotes the bandgap order.^[^ ^11^, ^12^, ^13^ ^]^

**Figure 2:**
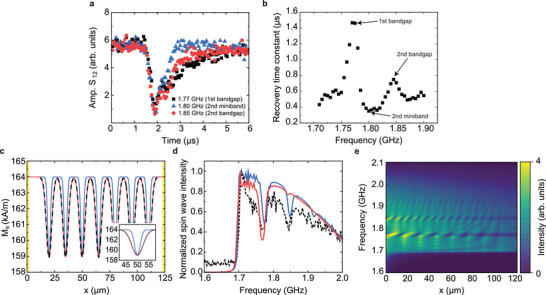
Analysis of temporal response and micromagnetic simulations. a) Spin‐wave transmission signal at selected frequencies for the *a*15*w*2.5 structure, extracted along horizontal lines in Figure 1d. A 500 ns laser pulse is applied at *t* = 1.5 µs. b) Recovery time constant (*τ*) as a function of frequency, obtained by fitting the post‐laser‐pulse evolution of the *S*_12_ signal to an exponential function. c) Spatial distribution of *M*_*s*_ used in micromagnetic simulations. The red curve shows a Gaussian fit to the black dashed line, which represents the *M*_*s*_ profile extracted from thermoplasmonic heating simulations (Figure 1c) and the calibrated dependence of *M*_*s*_ on temperature (Figure S9, Supporting Information). The blue curve corresponds to a Gaussian with the same center but a 50% reduced width. d) Simulated (solid red and blue lines) and measured (dashed black line) spin‐wave transmission spectra at Δ*t* = 0.7 µs for the *a*15*w*2.5 structure with *N* = 7 plasmonic stripes. The red and blue spectra are obtained using the corresponding *M*_*s*_ profiles shown in (c). Changes in the *M*_*s*_ modulation width affect the bandgap frequencies and depths. e) Simulated spatial distribution of spin‐wave intensity for the same structure at Δ*t* = 0.7 µs (using the blue *M*_*s*_ profile from (c)), demonstrating Bragg reflection and bandgap formation.

We now analyze the temporal response of the spin‐wave transmission signal in the *a*15*w*2.5 structure to a 500 ns laser pulse at three distinct frequencies: 1.77 GHz (first bandgap), 1.80 GHz (second miniband), and 1.85 GHz (second bandgap). **Figure** **2**a presents the recorded *S*_12_ scattering parameter at these frequencies, with the laser pulse activating at *t* = 1.5 µs. Upon laser illumination, the spin‐wave signal is suppressed across all frequencies, with a maximum attenuation of approximately 20 dB (corresponding to an amplitude ratio *S*_12_(laser on)/*S*_12_(laser off) of 0.1) observed at the end of the 500 ns laser pulse (*t* = 2.0 µs). After the laser switches off at *t* = 2.0 µs, the spin‐wave transmission signal begins to recover (Figure 2a). The recovery rate depends on the spin‐wave frequency, as illustrated in Figure 2b. The recovery follows an exponential trend described by: *S*_12_(*t*) = *S*_12_(*t* = 10) − (*S*_12_(*t* = 10) − *S*_12_(*t* = 2.0))exp(− *t*/*τ*), where *τ* is the recovery time constant (Figure S4, Supporting Information). At the first bandgap frequency, *τ* peaks at 1.45 µs, while it reaches 0.75 µs for the second bandgap and drops to 0.35 µs within the second miniband. These frequency‐dependent recovery times lead to the formation of bandgaps in the spin‐wave transmission spectrum shortly after the laser pulse. The bandgaps persist up to approximately Δ*t* = 1.0 µs (second bandgap) and Δ*t* = 3.0 µs (first bandgap). Similar results are observed for magnonic‐plasmonic metamaterials with different stripe periods (Figure S5, Supporting Information).

Figures 1d,e and 2a,b reveal two competing mechanisms that govern the spin‐wave transmission spectra following laser irradiation. Immediately after the laser pulse, elevated temperatures beneath the plasmonic stripes lead to a strong, broadband suppression of spin‐wave transmission. After a brief delay, however, well‐defined bandgaps emerge as the amplitude of the periodic temperature modulation in the YIG film diminishes. The initial strong suppression likely results from pronounced temperature gradients along the spin‐wave propagation direction (*x*‐axis). Supporting this interpretation, we observe that increasing the stripe width delays the onset of bandgap formation (Figure S6, Supporting Information). Thermoplasmonic heating simulations (Figure S7, Supporting Information) reveal that both the peak temperature and the cooling time increase with stripe width, indicating stronger localized heating and more persistent temperature gradients. These effects prolong the suppression period and enhance broadband attenuation of the spin‐wave signal at small Δ*t*. To further substantiate this mechanism, we performed propagating spin‐wave spectroscopy on a single plasmonic stripe (Figure S8, Supporting Information). The results confirm that even isolated stripes can induce broadband suppression shortly after laser excitation. We attribute this effect to a spin Seebeck‐driven spin‐transfer thermal torque acting on the magnetization in the presence of a strong temperature gradient.^[^ ^34^, ^35^ ^]^ When the gradient is sufficiently large, this torque dominates the spin‐wave dynamics and inhibits propagation. As the gradient relaxes during the cooling phase, the suppression weakens, allowing Bragg reflection to emerge and drive the formation of magnonic bandgaps.

The formation of bandgaps during cooling is attributed to a periodic modulation of the saturation magnetization (*M*_*s*_) in the YIG film, consistent with micromagnetic simulations (Figure 2c–e). To quantify the reduction in *M*_*s*_ beneath the plasmonic stripes, we measured FMR spectra using a suspended omega antenna while heating the sample on a ceramic heater (Figure S9a,b, Supporting Information). For instance, at 100 °C, the estimated temperature of the YIG film at the end of a 500 ns laser pulse (Figure 1c), *M*_*s*_ decreases to 136 kA/m, representing a 17% reduction from its room‐temperature value of 164 kA/m. By Δ*t* = 0.7 µs, the temperature beneath the stripes cools to approximately 35 °C, and *M*_*s*_ recovers to 159 kA/m (a 3% reduction). The calibration of *M*_*s*_(*T*) is further validated by vibrating sample magnetometry (VSM) measurements (Figure S9c,d, Supporting Information). We incorporated the resulting periodic modulation of *M*_*s*_ into micromagnetic simulations using the MuMax3 software package^[^ ^36^ ^]^ (see Experimental Section). The black dashed curve in Figure 2c shows the *M*_*s*_ profile at Δ*t* = 0.7 µs derived from thermal modeling, while the red curve represents a Gaussian fit used in the simulations. The simulated spin‐wave transmission spectrum (Figure 2d, red line) reproduces key experimental features, including the formation of bandgaps and low‐loss minibands. A closer match with experimental data is achieved when the width of the *M*_*s*_ profile is reduced by 50% (Figure 2d, blue line), suggesting that thermal diffusion in the experimental YIG film is somewhat lower than predicted by the model. Results for other Δ*t* values, shown in Figure S10 (Supporting Information), further support that the periodic modulation of *M*_*s*_ induced by thermoplasmonic heating is responsible for the formation of multiple laser‐induced magnonic bandgaps.

**Figure 3:**
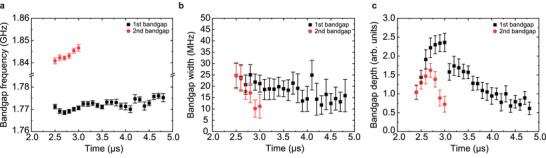
Parameters of laser‐induced bandgaps. a–c) Time evolution of the frequencies, widths, and depths of the first and second bandgaps in the spin‐wave transmission spectra of the *a*15*w*2.5 structure after the deactivation of a 500 ns laser pulse (*P* = 16.6 W) at *t* = 2.0 µs. The widths and depths shown in (b) and (c) are extracted by fitting the bandgaps in the spectra with a Lorentzian function (Figure S11, Supporting Information).

**Figure** **3** presents the time evolution of the frequencies, widths, and depths of the first and second bandgaps following the 500 ns laser pulse. The bandgap widths and depths are extracted from time‐resolved spin‐wave transmission spectra by fitting each bandgap with a Lorentzian function, with the gap center approximated by the Lorentzian minimum (Figure S11, Supporting Information). As the system cools, the bandgap frequencies gradually shift upward (Figure 3a), which we attribute to the recovery of *M*_*s*_. Simultaneously, the bandgap widths decrease over time (Figure 3b), indicating a reduction in magnetic contrast within the magnonic crystal.^[^ ^37^ ^]^ This behavior is driven by the decreasing modulation of *M*_*s*_ across the YIG film as the temperature returns to equilibrium. The evolution of the bandgap depths (Figure 3c), defined as the difference between the signal just outside the bandgap and the *S*_12_ amplitude minimum in the gap, is non‐monotonic. Notably, the first bandgap persists longer than the second, consistent with the recovery time constants shown in Figure 2b. These trends are qualitatively reproduced by micromagnetic simulations (Figure S12, Supporting Information), which show that broadening of the *M*_*s*_ profile due to lateral heat diffusion initially enhances the bandgap depths before they eventually decrease. However, the enhancement is much more pronounced and longer‐lasting for the first bandgap than for higher‐order bandgaps, with the latter disappearing earlier while the first bandgap remains. Consequently, multiple bandgaps are only observed when the *M*_*s*_ profile is sufficiently narrow.

The data presented thus far describe the metamaterial's response to individual laser pulses. Alternatively, high‐frequency laser pulsing can be employed. In this regime, localized thermoplasmonic heating during each pulse is balanced by cooling between pulses, potentially establishing a quasi‐static magnetization profile and a corresponding magnonic band structure while pulsing is ongoing. We investigated this scenario across a range of pulsing frequencies and laser powers, with the results summarized in Figure S13 (Supporting Information). However, high‐frequency pulsing was found to reduce the overall spin‐wave transmission intensity, leading to shallower bandgaps or, in some cases, their complete suppression. Importantly, after prolonged high‐frequency pulsing, the system's response to individual laser pulses could still be fully reproduced, demonstrating the high durability and reversibility of thermoplasmonic control over spin‐wave transport.

**Figure 4:**
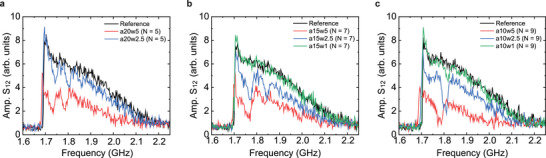
Influence of plasmonic parameters on spin‐wave transport. a–c) Spin‐wave transmission spectra for different magnonic‐plasmonic metamaterials, recorded 0.7 µs after the deactivation of a 500 ns laser pulse (*P* = 16.6 W). The spectra illustrate the effects of varying the plasmonic stripe width (*w*), stripe array period (*a*), and the number of stripes (*N*).

We now investigate the effects of plasmonic stripe width (*w*), stripe array period (*a*), and the number of stripes (*N*) on spin‐wave transport. **Figure** **4** shows the spin‐wave transmission spectra for various magnonic‐plasmonic metamaterials, recorded 0.7 µs after the deactivation of a 500 ns laser pulse. The spectra exhibit bandgaps, each corresponding to a Bragg scattering condition (*k* = *n* *π*/*a*, see Figure 1e,f), along with allowed minibands. These features evolve systematically with changes in the plasmonic parameters. Decreasing the stripe array period (*a*) enlarges the Bragg wave vectors, causing the bandgaps to shift to higher frequencies while the spacing between them widens. Increasing the plasmonic stripe width (*w*) enhances thermoplasmonic heating in the YIG film (Figure S7, Supporting Information), which suppresses the spin‐wave signal across the entire spectrum and shifts the bandgaps to lower frequencies due to a more pronounced reduction in *M*_*s*_. Remarkably, even the narrowest stripes (*w* = 1 µm), consisting of only three nanodisks along the spin‐wave propagation direction, are sufficient to open a bandgap in the spin‐wave transmission spectrum under laser illumination (green curve in Figure 4c). Increasing the number of stripes (*N*) further enhances the efficiency of Bragg scattering, resulting in deeper and wider bandgaps, consistent with previous studies on magnonic crystals.^[^ ^11^, ^12^, ^13^ ^]^ Time‐resolved spin‐wave spectroscopy maps, similar to the one shown in Figure 1d, are provided in Figure S6 (Supporting Information) for all hybrid metamaterials.

**Figure 5:**
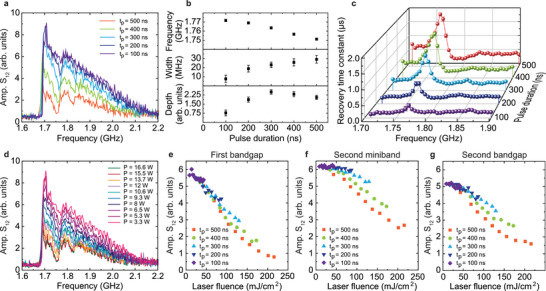
Dependence of spin‐wave transport on pulse duration and laser power. a) Spin‐wave transmission spectra for the *a*15*w*5 structure (*N* = 7) recorded 500 ns after illumination by single laser pulses of varying durations (*t*_*p*_) with a constant laser power of 16.6 W. b) Variation in the first bandgap frequency, width, and depth as a function of pulse duration, derived from the data shown in (a). c) Recovery time constant (*τ*) as a function of frequency for different pulse durations, obtained by fitting the post‐laser‐pulse evolution of the *S*_12_ signal to an exponential function. d) Spin‐wave transmission spectra for the same structure recorded 500 ns after illumination by single laser pulses with varying power (*P*) and a fixed duration of 500 ns. e–g) Spin‐wave transmission signal in the fist bandgap, the second miniband, and the second bandgap, respectively, plotted against laser fluence (*P* × *t*_*p*_/*A*, where *A* is the area of the laser spot), for all combinations of laser power and pulse duration.

Various laser parameters, such as pulse duration and power, provide versatile control over the spatiotemporal manipulation of propagating spin waves in the hybrid magnonic‐plasmonic metamaterials. Thus far, our focus has been on experiments conducted with a 500 ns pulse duration and a power of 16.6 W, the maximum laser output, corresponding to a power density of 4.3 mW/µm^2^ on the plasmonic nanodisk array. **Figure** **5**a,d illustrates the effect of pulse duration and power on spin‐wave transmission in the *a*15*w*5 structure, with all measurements recorded 0.5 µs after the laser pulse. Spectra obtained for pulse durations ranging from 100 to 500 ns (at *P* = 16.6 W) consistently exhibit bandgaps (Figure 5a), although their frequencies, widths, depths, and the overall transmission intensity are highly sensitive to pulse duration. Longer pulses deliver more energy to the YIG film via thermoplasmonic heating, leading to greater temperature variations and a stronger periodic modulation of *M*_*s*_. Consequently, the first bandgap becomes wider, deeper, and shifts to lower frequencies with increasing pulse duration (Figure 5b). Additionally, longer pulses extend the duration of the optically induced magnonic band structure, as reflected in the extracted signal recovery time constants shown in Figure 5c.

Figure 5d presents the dependence of spin‐wave transmission on laser power for a fixed pulse duration of 500 ns. Additional spin‐wave spectra for various combinations of laser power and pulse duration are provided in Figure S14 (Supporting Information). As the laser power decreases, reducing thermoplasmonic heating, the transmission signal at Δ*t* = 0.5 µs increases. However, both the first and second bandgaps persist under all conditions. To assess the influence of temporal dynamics on the magnonic band structure, we plot the transmission signal within the first bandgap, second miniband, and second bandgap as a function of laser fluence in Figure 5e–g for all combinations of laser power and pulse duration. At low laser fluences, the data points collapse onto a single curve, with slight deviations emerging at higher fluences. This behavior suggests that the total energy absorbed by the system predominantly governs the spatiotemporal control of spin‐wave transport.

## 3 Conclusion

In summary, we have demonstrated a novel hybrid magnonic‐plasmonic metamaterial designed for spatiotemporal control of spin‐wave transport. By exploiting surface lattice resonance modes in a plasmonic stripe pattern, we achieved dynamic modulation of the saturation magnetization in an YIG film via thermoplasmonic heating. This mechanism enables the temporary formation of a tunable magnonic band structure characterized by multiple bandgaps and minibands, created through optical excitation with laser pulses as short as 100 ns and stripe widths as small as 1 µm. Our findings reveal that the suppression of spin‐wave transmission within the bandgaps is governed by key parameters including pulse duration, laser power, and cooling dynamics following laser deactivation. These results underscore the potential for precise, on‐demand control of spin‐wave propagation, opening new avenues for the development of advanced magnonic devices.

Beyond demonstrating the fundamental principles of this hybrid system, our work lays the foundation for its application in magnonic computing and signal processing. The capability to actively control spin waves in both spatial and temporal domains offers a versatile and scalable platform for encoding, transmitting, and processing analog and digital information. Future efforts could focus on optimizing device geometries and scaling the approach down to nanoscale dimensions. Such advancements would further position hybrid magnonic‐plasmonic systems as a compelling platform for next‐generation information technologies.

## 4 Experimental Section

### Sample Fabrication

An LPE‐grown YIG film was used on a GGG(111) substrate obtained from Matesy GmbH.^[^ ^38^, ^39^ ^]^ The 80‐nm‐thick YIG film exhibited a Gilbert damping parameter of 7 × 10^−4^ (see Figure S15, Supporting Information). Plasmonic nanodisk arrays were patterned onto the YIG film using electron‐beam lithography. Following the development of the polymethyl methacrylate (PMMA) resist, a 3 nm Ti/50 nm Au layer was deposited via electron‐beam evaporation and processed using a lift‐off technique in hot acetone. The resulting nanodisk arrays, with 180‐nm‐diameter disks and 400 nm array periods, were designed to enhance light absorption at 915 nm by leveraging SLR mode excitation (Figure S1, Supporting Information). The nanodisk arrays were configured into regular stripe patterns to locally modulate the *M*_*s*_ in YIG through thermoplasmonic heating. Stripe widths (*w*) ranged from 1 to 5 µm, while stripe periods (*a*) varied from 10 to 20 µm. Metamaterials consisting of five to nine stripes were fabricated to investigate the optical control of spin‐wave transport. Parallel microwave antennas for spin‐wave excitation and detection were fabricated on both sides of the plasmonic structures using maskless photolithography and lift‐off processes. These antennas, made from 3 nm Ti/120 nm Au, were 6 µm wide, 50 µm long, and separated by 125 µm.

### Optical Transmittance Measurements

The SLR modes of the plasmonic nanodisk arrays, patterned into periodic stripe patterns on the YIG film, were characterized using an optical spectrometer. The setup included a supercontinuum laser (NKT SuperK EXW‐12) equipped with tunable acousto‐optical filters, along with polarizing and focusing optics, and a broadband balanced photodetector. The laser beam was focused onto the sample at normal incidence, with the wavelength tuned between 500 and 1100 nm. Optical transmittance spectra were recorded and normalized to a reference spectrum obtained from the bare YIG film, allowing the optical response of the plasmonic arrays to be isolated.

### Time‐Resolved Propagating Spin‐Wave Spectroscopy

Time‐resolved spin‐wave transmission experiments under laser illumination were conducted using a home‐built probe station. The setup included a two‐port VNA (Agilent N5222A), a waveform generator (Agilent 33220A), a 915 nm pulsed laser (AeroDiode 915LD‐4‐2), and a quadrupole electromagnet. Spin‐wave transport between the antennas was characterized by measuring the *S*_12_ scattering parameter, with the microwave excitation power set to −20 dBm to avoid nonlinear spin‐wave excitation. A magnetic bias field of 16 mT, aligned parallel to the microwave antennas, was applied to establish spin‐wave propagation in the Damon‐Eshbach geometry. Time‐resolved measurements were enabled by synchronizing the VNA and pulsed laser via a trigger signal from the waveform generator. The trigger signal defined the time‐zero reference, with the laser pulse delayed by 1.5 µs relative to the trigger's leading edge. Spin‐wave transmission signals were acquired in a pump‐probe fashion by sweeping the VNA's internal measurement delay relative to the trigger. In this technique, the time resolution was determined by the selected frequency bandwidth of the VNA, while the excitation frequency could be precisely controlled through the VNA settings. To enhance the signal‐to‐noise ratio, 5 to 10 averages were performed per delay step, with the VNA's intermediate frequency bandwidth set to 1.5 MHz. Time‐resolved maps of spin‐wave transmission spectra (Figure 1d; Figure S11, Supporting Information) were obtained by incrementally increasing the excitation frequency in 2.5 MHz steps. The laser beam, focused onto the plasmonic arrays with a 70 µm spot diameter, was operated at pulse durations of 100 to 500 ns and power levels from 3.3 to 16.6 W.

### Modeling of Thermoplasmonic Heating

Thermoplasmonic heating effects were modeled using Ansys Lumerical finite‐difference time‐domain (FDTD) and HEAT solvers. FDTD simulations determined the power absorbed by a plasmonic array on an YIG film with a thick GGG substrate. The simulation area, measuring 400 × 400 nm^2^ (single unit cell), used periodic boundary conditions along the *x‐* and *y*‐axes. The nanodisk diameter (180 nm) and array period (400 nm) matched the experimental dimensions, with the nanodisks surrounded by air. To suppress reflections from the edges of the simulation area, perfectly matched layer (PML) absorbing boundaries were implemented along the *z*‐axis, positioned far from both the nanodisk and the YIG film. The system was illuminated with a normally incident, linearly polarized plane wave at a wavelength of 915 nm. An input optical power of 0.7 mW per unit cell was used, corresponding to *P* = 16.6 W for the 70 µm laser spot used in experiments. Power absorption by a single unit cell was extracted using an internal library. These results were imported into the HEAT solver for thermal modeling.

Thermal simulations were performed in a 15 µm × 400 nm area with a single 2.5‐µm‐wide stripe and 2D periodic boundary conditions were used to emulate the *a*15*w*2.5 stripe pattern. The heat source was activated for 500 ns, and heat losses to air were incorporated via convective boundary conditions. The thermal conductivities of the YIG film and GGG substrate were set to *k*_*YIG*_ = 6 W/mK and *k*_*GGG*_ = 8 W/mK,^[^ ^40^ ^]^ while their optical constants were taken from Ref. [41]. Temperature profiles were captured in the *xy*‐ and *xz*‐planes to evaluate the time evolution of thermoplasmonic heating during a laser pulse (Figure 1c).

### Micromagnetic Simulations

Spin‐wave transport in hybrid magnonic‐plasmonic metamaterials was modeled using MuMax3.^[^ ^36^ ^]^ The simulation area, measuring 700 × 10 µm^2^, was discretized into 10 × 10 × 80 nm^3^ cells, with 1D periodic boundary conditions along the *y*‐axis. The following YIG parameters were used: saturation magnetization *M*_*s*_ = 164 kA/m, exchange constant *A*_*ex*_ = 3.1 pJ/m, and magnetic damping parameter α = 7 × 10^−4^ (see Figure S15, Supporting Information). High‐damping boundary conditions suppressed edge reflections, and a 16 mT bias field along the *y*‐axis replicated experimental conditions.

Periodic thermoplasmonic heating effects were incorporated by reducing the *M*_*s*_ beneath the plasmonic stripes based on temperature‐dependent profiles obtained from thermoplasmonic simulations (Figure 1c). The spatial profile of *M*_*s*_ was modeled as a Gaussian function:

(1) $$M_{s}\left(x\right)=M_{s,RT}-\frac{A}{w\sqrt{\pi /2}}exp\left(-2\frac{x^{2}}{w^{2}}\right)$$

where *M*_*s*, *RT*_, is the room‐temperature saturation magnetization, *A* is the modulation amplitude, and *w* is the width of the Gaussian profile. The modulation of *M*_*s*_ was determined using thermal simulation results and calibrated *M*_*s*_‐versus‐temperature data (Figure S9, Supporting Information).

Spin waves were excited at *x* = 0 µm using 1 mT sinc‐function magnetic field pulses with a 2.2 GHz cut‐off frequency. The time evolution of *m*_*x*_ (the x‐component of magnetization) was recorded over 300 ns. Spin‐wave intensity maps (Figure 2e) were generated by Fourier transforming *m*_*x*_ on a cell‐by‐cell basis, and transmission spectra (Figure 2d; Figure S10, Supporting Information) were extracted from spin‐wave intensities at *x* = 125 µm, matching the experimental detection antenna location.

## Conflict of Interest

The authors declare no competing interests.

## Supporting information

Supporting Information

## Data Availability

The data that support the findings of this study are available from the corresponding author upon reasonable request.
