# Supplementary material for: Dynamic Magnonic Crystals Based on Spatiotemporal Plasmon Excitation
Source: Adv Mater. 2025 Jun 3;37(33):2502474. doi: 10.1002/adma.202502474 (PMC12369674; doi:10.1002/adma.202502474)
Supplement: Supplementary file 1 — Supporting Information [file ADMA-37-2502474-s001.pdf]

ADVANCED MATERIALS

Supporting Information

for *Adv. Mater.*, DOI 10.1002/adma.202502474

Dynamic Magnonic Crystals Based on Spatiotemporal Plasmon Excitation

*Nikolai Kuznetsov, Huajun Qin, Lukáš Flajšman and Sebastiaan van Dijken**

Dynamic Magnonic Crystals Based on Spatiotemporal Plasmon Excitation

*Nikolai Kuznetsov Huajun Qin Lukáš Flajšman Sebastiaan van Dijken**

N. Kuznetsov, Dr. Flajšman, Prof. S. van Dijken
NanoSpin, Department of Applied Physics, Aalto University School of Science, Aalto FI-00076, Finland
Email Address: sebastiaan.van.dijken@aalto.fi

Prof. H. Qin
School of Physics and Technology, Wuhan University, Wuhan 430072, China
Wuhan Institute of Quantum Technology, Wuhan 430206, China

Keywords: *Magnonics, Plasmonics, Metamaterials, Dynamic Magnonic Crystals*

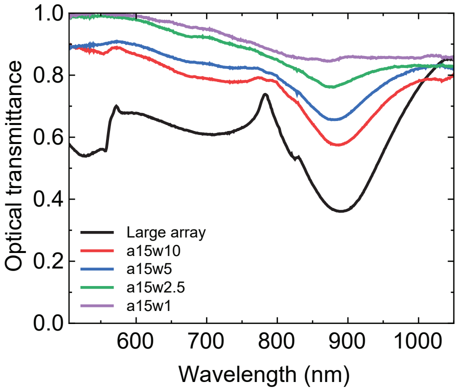

Figure 1: Optical transmittance spectra of regular Au plasmonic stripe patterns ($N = 7$) on an 80-nm-thick YIG film. Each stripe consists of Au nanodisk arrays with a fixed disk diameter (d) of 180 nm and a constant array period (p) of 400 nm. For comparison, the transmittance spectrum of a larger, 250×250 μm^2 nanodisk array with identical d and p values is also shown. All spectra exhibit enhanced optical absorption near 900 nm, corresponding to the excitation of a surface lattice resonance (SLR) mode. The reduction in optical transmittance for narrower stripes is attributed to a lower nanodisk filling factor within the laser spot.

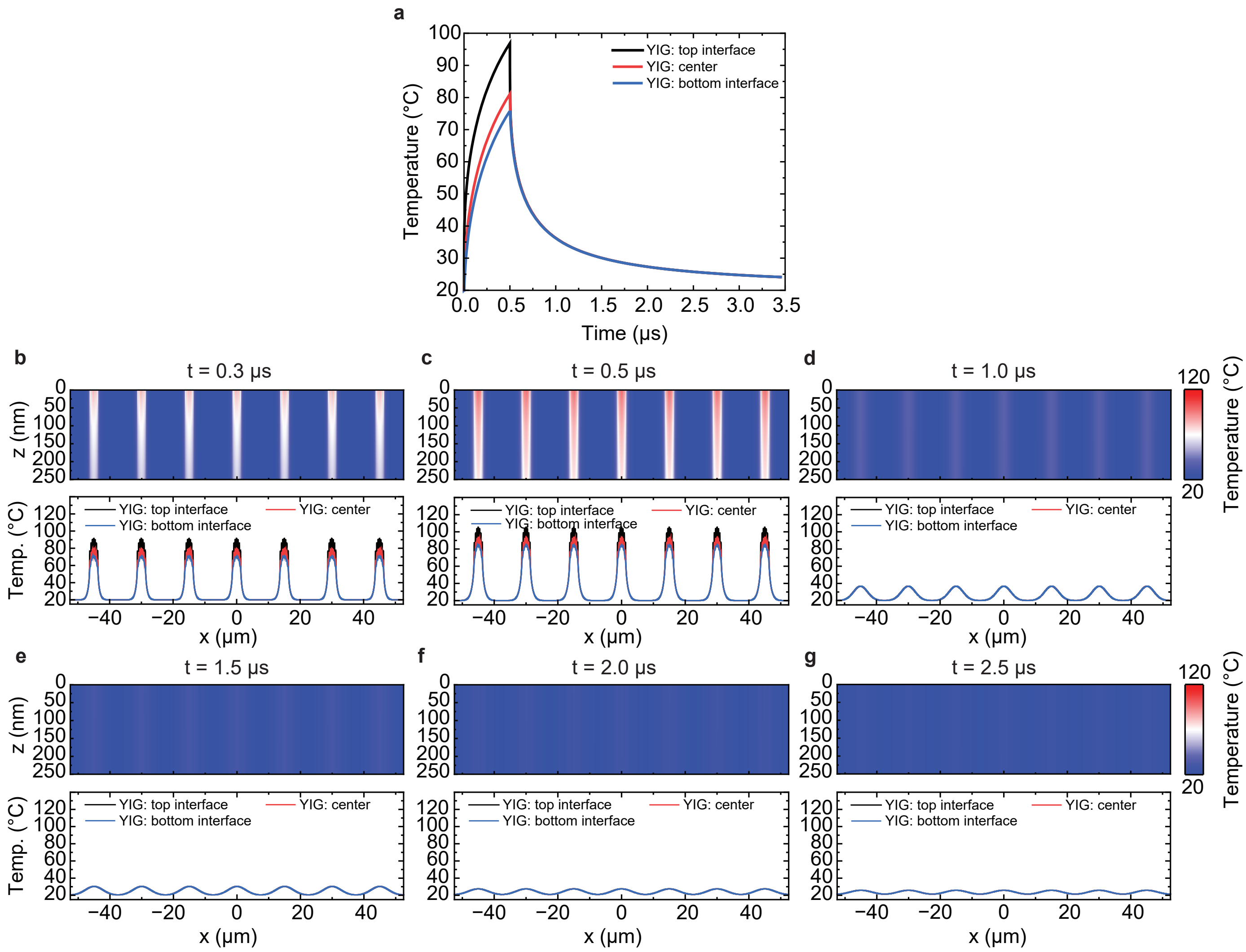

Figure 2: Modeling of thermoplasmonic heating in the $a15w2.5$ structure under a 500 ns laser pulse. The normally incident plane wave has a power density of 4.3 mW$/\mu$m^2, consistent with a laser power $P = 16.6$ W and a laser spot diameter of 70 μm in experiments. a) Simulated temperature evolution, showing an increase when the laser pulse is turned on and subsequent cooling after the pulse is turned off. Data are presented for the top interface, center, and bottom interface of the YIG film. b-g) Thermal profiles in the xz-plane of the YIG film at selected times (t) after the laser pulse is turned on (top panels) and line scans of the temperature distribution in the YIG film for the corresponding t (bottom panels). Note that data shown in Figure 1c of the main manuscript are plotted for various Δt, the time after the pulse is turned off.

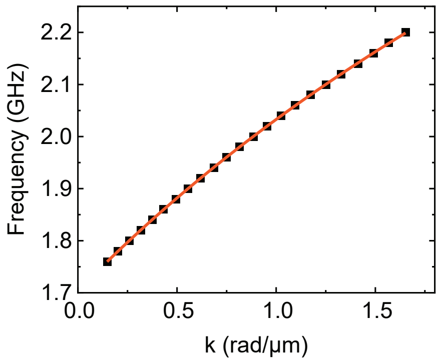

Figure 3: Spin-wave dispersion extracted from SNS-MOKE microscopy measurements at different frequencies (symbols) and a fit to the data for Damon-Eshbach spin waves [1, 2].

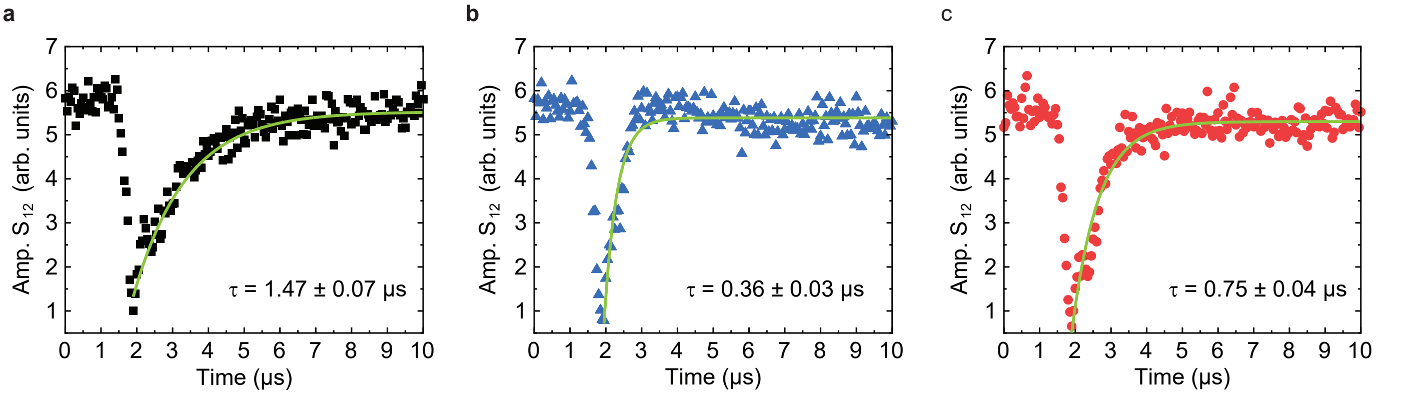

Figure 4: Time evolution of the spin-wave transmission signal for the $a15w2.5$ structure at (a) 1.77 GHz (first bandgap), (b) 1.80 GHz (second miniband), and (c) 1.85 GHz (second bandgap). A 500 ns laser pulse with $P = 16.6$ W is applied at $t = 1.5$ μs. The recovery time constants (τ), obtained from exponential fits to the data recorded after the laser is turned off (green lines), are indicated in each graph.

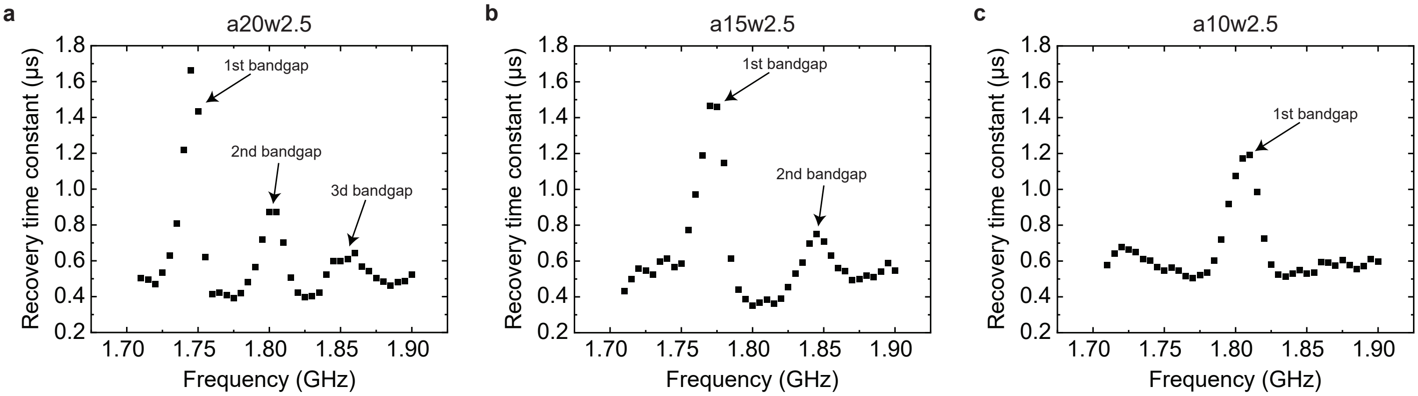

Figure 5: Recovery time constant (τ) as a function of frequency for three metamaterial structures: $a20w2.5$, $a15w2.5$, and $a10w2.5$. Values of τ are obtained by fitting the post-laser-pulse evolution of the S_{12} signal to an exponential function.

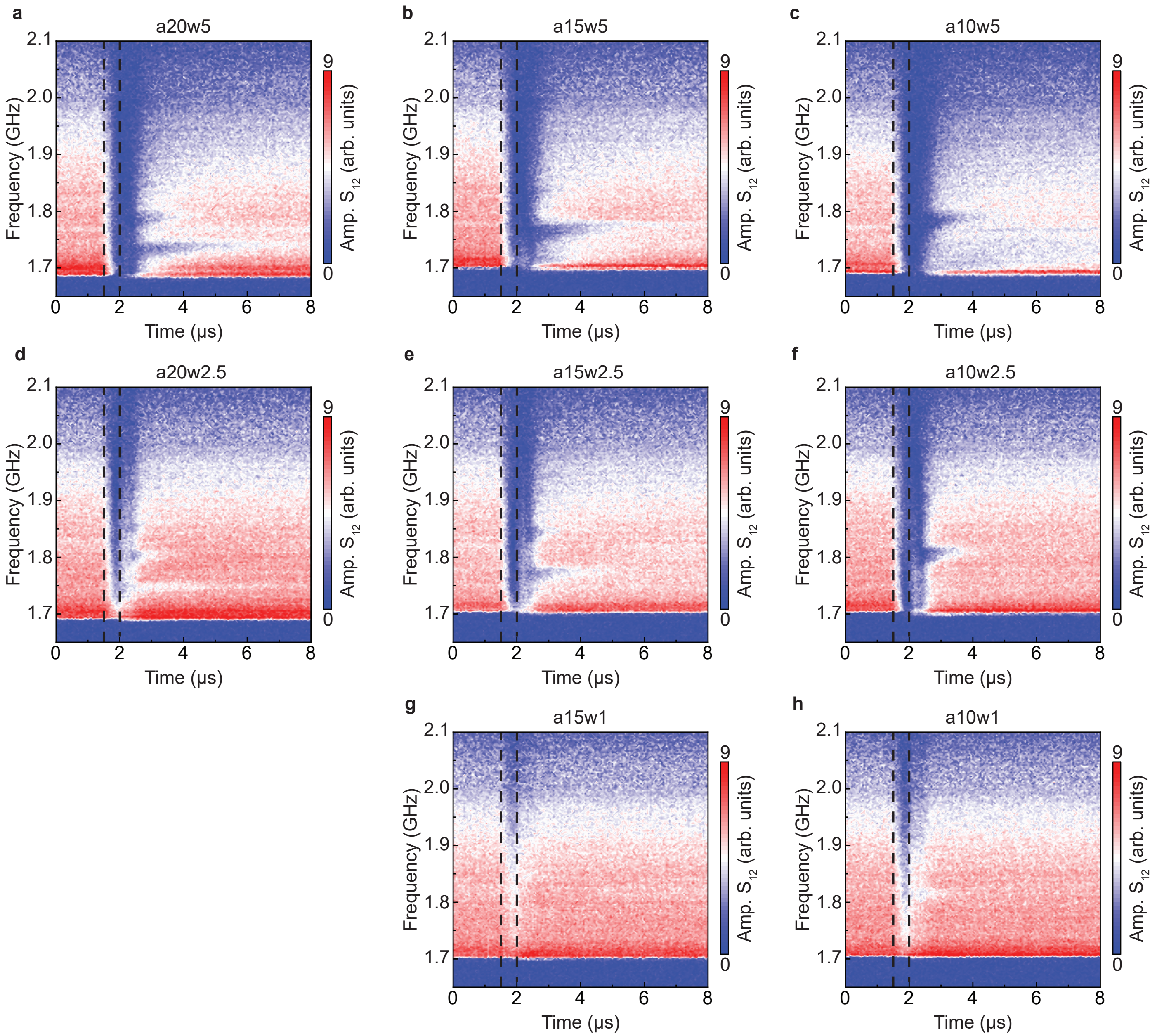

Figure 6: Time-resolved maps of spin-wave transmission spectra for different magnonic-plasmonic metamaterials, with the 500 ns laser pulse (P = 16.6 W) activated at t = 1.5 μs. The vertical dashed lines mark the laser pulse activation and deactivation times.

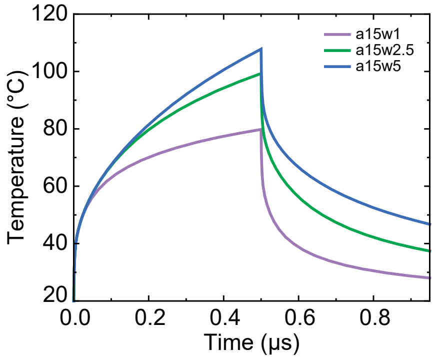

Figure 7: Modeling of thermoplasmonic heating in the $a15w1$, $a15w2.5$, and $a15w5$ structures under a 500 ns laser pulse. The normally incident plane wave has a power density of 4.3 mW/μm^2, consistent with a laser power $P = 16.6$ W and a laser spot diameter of 70 μm in experiments. The peak temperature beneath the plasmonic stripes and the cooling time increase with stripe width.

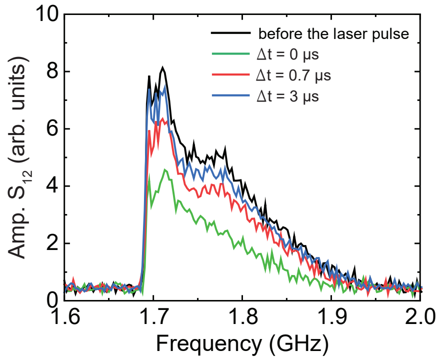

Figure 8: Spin-wave transmission spectra for a single 10-μm-wide plasmonic stripe, recorded at different times after a 500 ns laser pulse ($P = 16.6$ W) is switched off. The stripe consists of an array of Au nanodisks (diameter $d = 180$ nm, period $p = 400$ nm) patterned on top of the YIG film. The stripe is positioned between two parallel microwave antennas that are separated by 200 μm.

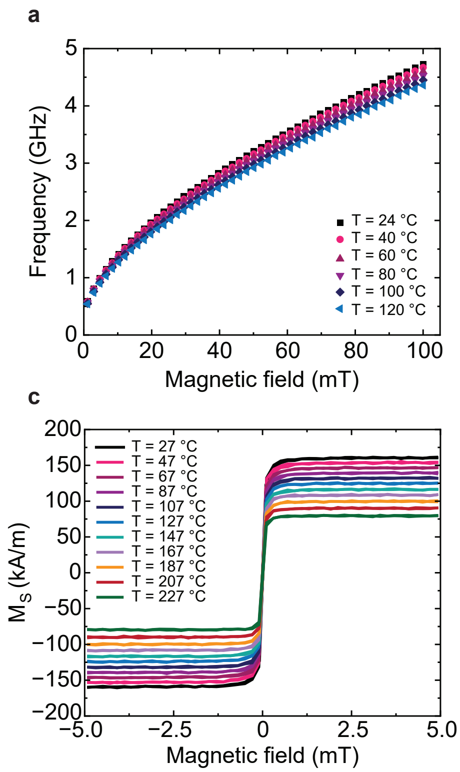

Figure 9: a) FMR frequency as a function of magnetic bias field measured at different temperatures. The FMR spectra were obtained using a suspended omega antenna while the sample was heated on a ceramic heater. b) Saturation magnetization of the 80-nm-thick YIG film as a function of temperature, extracted by fitting the data in (a) with the Kittel formula [3]. The temperature dependence of M_s is modeled as $M_s(T) = M_{s,RT} - \eta(T - T_{RT})$, with $\eta = 377\ \mathrm{AK^{-1}m^{-1}}$. c) VSM hysteresis loops recorded at different temperatures using a physical property measurement system (PPMS). d) Temperature dependence of M_s extracted from the data in (c). Both measurement methods reveal a nearly linear decrease in M_s with increasing temperature up to approximately 120 °C.

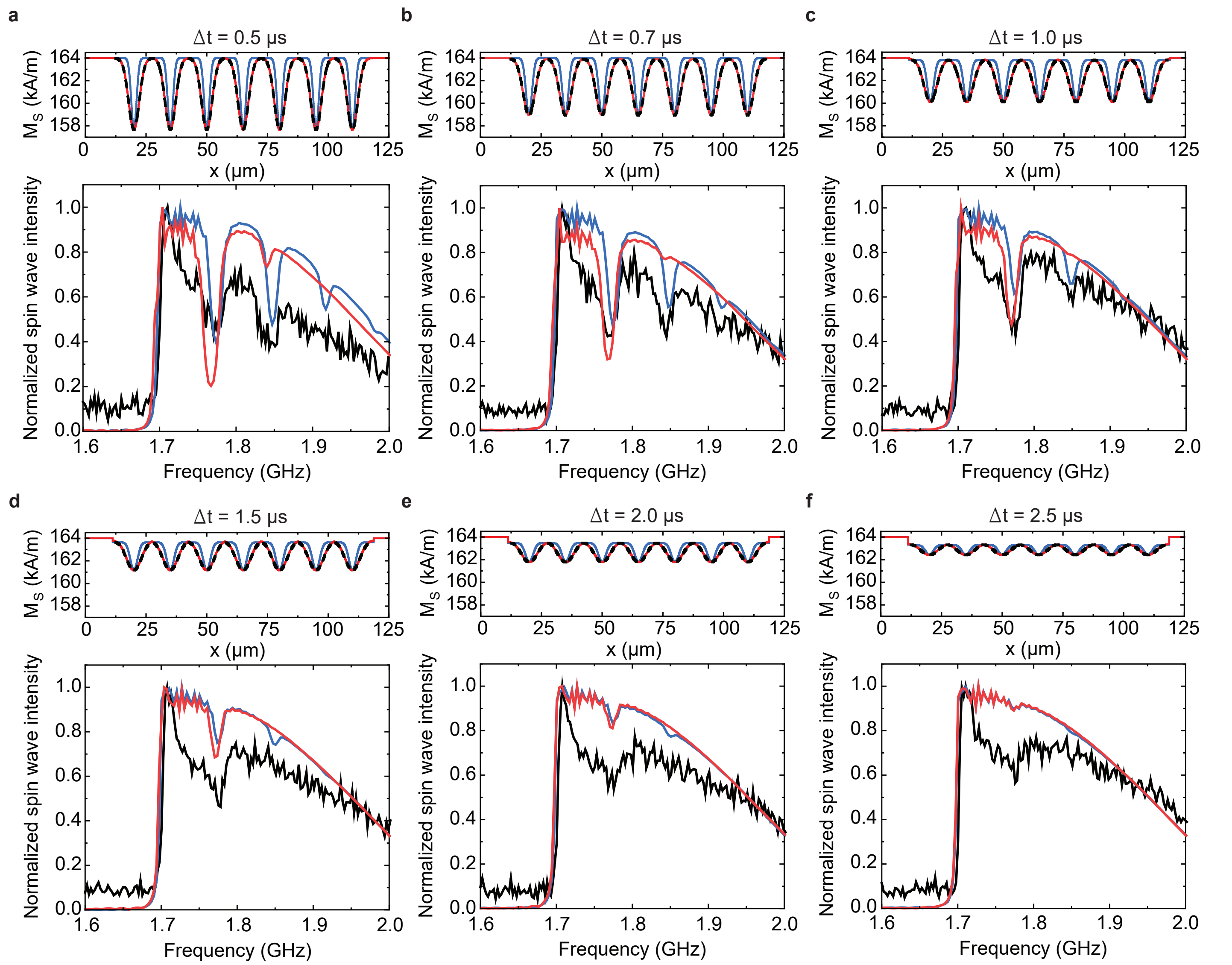

Figure 10: Simulated (red and blue curves) and measured (black curve) spin-wave transmission spectra for the $a15w2.5$ structure with $N = 7$ plasmonic stripes. Results are shown at different times (Δt) after a 500 ns laser pulse is turned off. The spatial distributions of M_s used in the simulations are shown in the upper panels. The red curve corresponds to a Gaussian fit to the M_s profile extracted from thermoplasmonic heating simulations and the calibrated temperature dependence of M_s (black curve). The blue curve represents a Gaussian profile centered at the same position but with a 50% reduced width.

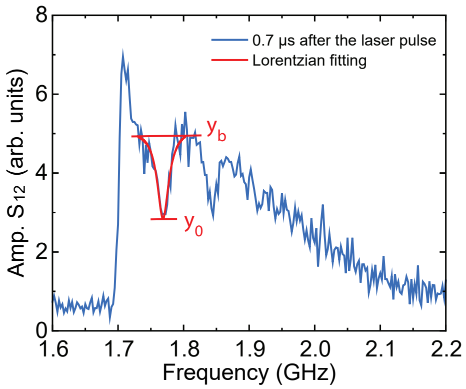

Figure 11: Illustration of method used to determine bandgap frequencies, widths, and depths from spin-wave transmission spectra. The example shows data for the $a15w2.5$ structure, recorded 0.7 μs after a 500 ns laser pulse is turned off. The first bandgap is modeled using a Lorentzian fit, allowing extraction of the approximate center frequency, width, and depth. The depth of the bandgap is defined as $y_b - y_0$ [4].

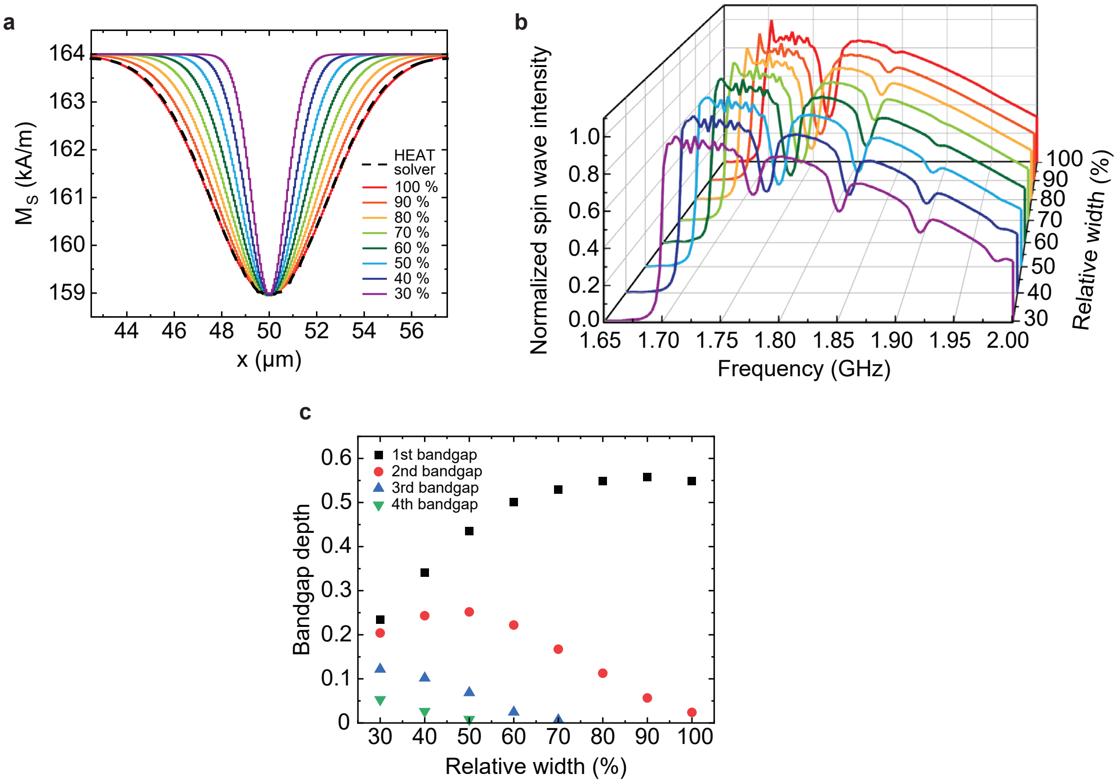

Figure 12: Micromagnetic simulations illustrating how broadening of the reduced M_s profile affects the bandgaps in spin-wave transmission spectra. a) Profiles of reduced M_s beneath the plasmonic stripes used in the simulations. The dashed black curve corresponds to the M_s profile derived from thermoplasmonic heating simulations and the calibrated temperature dependence of M_s at $\Delta t = 0.7$ μs. The red curve is a Gaussian fit to this result, while the other curves represent Gaussian profiles centered at the same position but with progressively reduced widths. b) Simulated spin-wave transmission spectra for the $a15w2.5$ structure with $N = 7$ plasmonic stripes, obtained using the M_s profiles defined in (a). c) Depths of the first, second, third, and fourth bandgaps plotted as a function of the M_s profile width. The results show that broadening of the M_s profile over time, due to lateral thermal diffusion, affects the bandgaps differently: higher-order bandgaps vanish more rapidly, while the first bandgap persists the longest, in qualitative agreement with experimental data shown in Figures 2b and 3c in the main manuscript.

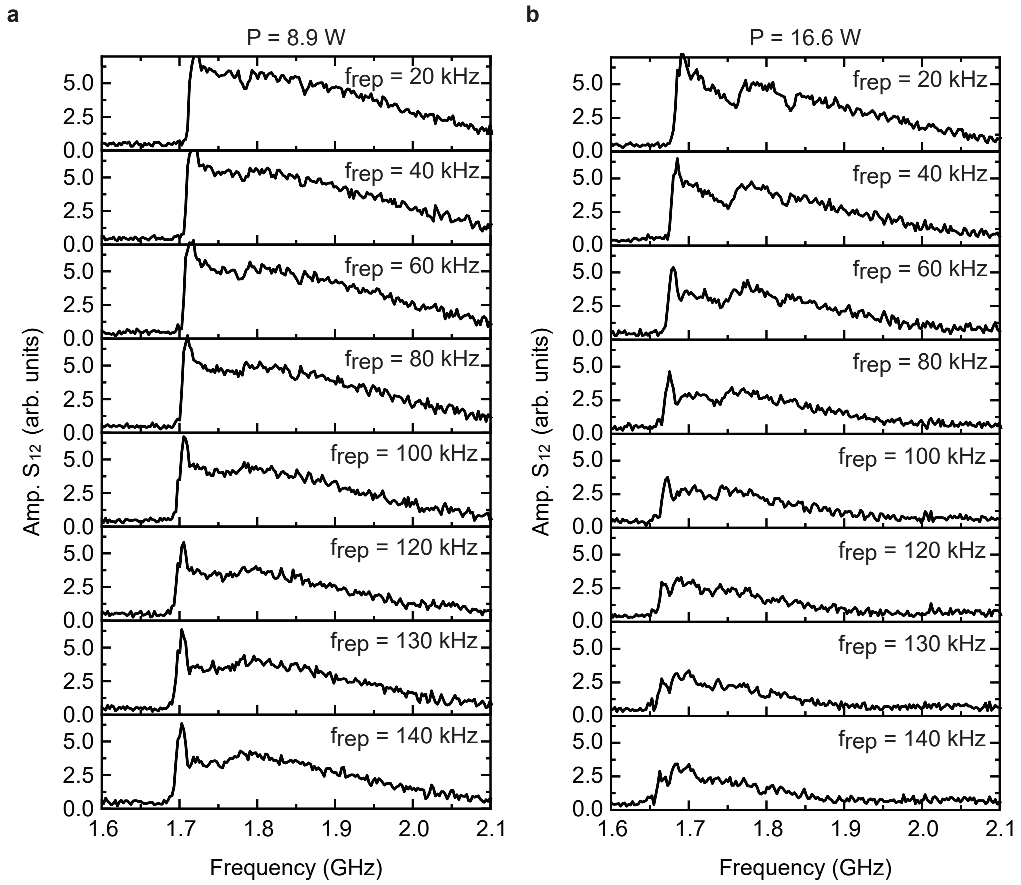

Figure 13: a,b) Spin-wave transmission spectra recorded on the $a15w2.5$ structure ($N = 7$) at different laser repetition rates (f_{rep}) and laser powers (P). The data were collected at a time delay $\Delta t = 0.7\ \mu$s with 500 ns laser pulses.

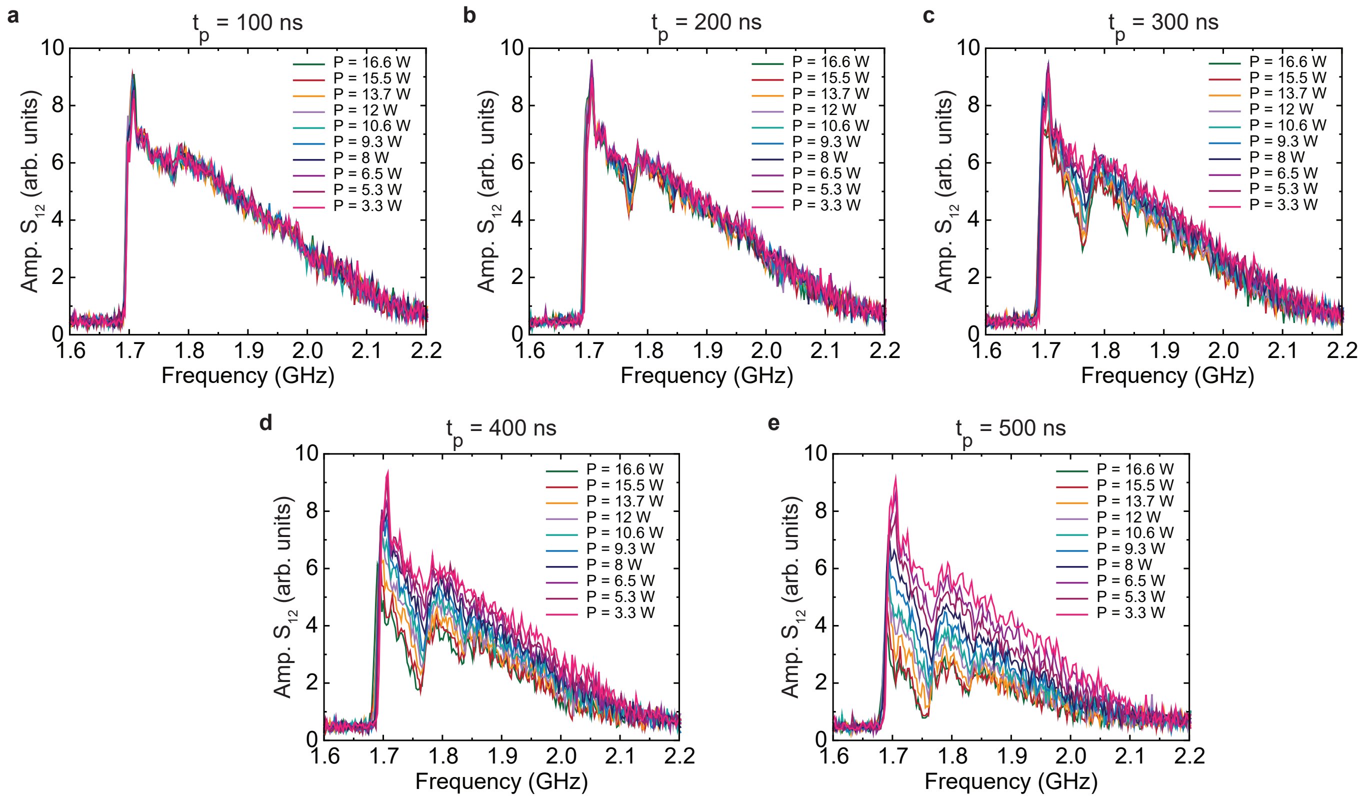

Figure 14: Dependence of spin-wave transport on pulse duration ($100 - 500$ ns) and laser power ($3.3 - 16.6$ W) for the $a15w5$ structure ($N = 7$) recorded 500 ns after laser illumination.

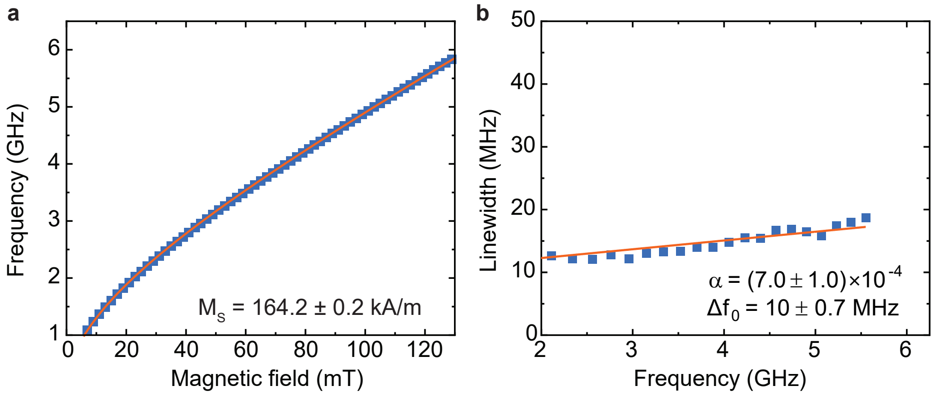

Figure 15: Magnetic properties of the 80-nm-thick YIG film. a) Frequency of the YIG FMR resonance as a function of the magnetic bias field, measured by placing the YIG film face-down onto a coplanar waveguide. The Kittel fit [3] to the experimental data (orange line) yields a saturation magnetization $M_s = 164$ kA/m. b) Linewidth of the YIG FMR resonance as a function of frequency. A linear fit to the data using $\Delta f = 2\alpha f + \Delta f_0$ gives a magnetic damping parameter $\alpha = 7 \times 10^{-4}$. The inhomogeneous linewidth broadening, Δf_0, is 10 MHz.

References

[1] B. Kalinikos, A. Slavin, *Journal of Physics C: Solid State Physics* **1986**, *19* 7013.

[2] D. D. Stancil, *Theory of Magnetostatic Waves*, Springer New York, NY, **1993**.

[3] C. Kittel, *Physical Review* **1948**, *73* 155.

[4] T. Goto, K. Shimada, Y. Nakamura, H. Uchida, M. Inoue, *Physical Review Applied* **2019**, *11* 014033.
